# Supplementary material for: An immunogenic model of KRAS-mutant lung cancer enables evaluation of targeted therapy and immunotherapy combinations
Source: Cancer Res. Author manuscript; Available in PMC 2022 Oct 5. (PMC7613674; doi:10.1158/0008-5472.CAN-22-0325)
Supplement: Supplementary Material [file EMS152609-supplement-Supplementary_Material.zip › supp_info_1 (2).pdf]

SUPPLEMENTARY INFORMATION

Boumelha, de Carné, Law et al.

Supplementary Methods

Rosa26::LSL-A3Bi knock-in construction

An intron-containing human *A3B* cDNA (*A3Bi*) (1) was amplified using 5'-**ACG-CGT-**ACC-ATG-AAT-CCA-CAG-A and 5'- **ACG-CGT-**tca-GTT-TCC-CTG-ATT-C (*Mlu*I sites in bold) and cloned into pJET2.1 (Clontech). The resulting cDNA was subcloned into a *Mlu*I-digested *Rosa26* gene targeting vector pROSA26-1 (gift from Philippe Soriano; Addgene #21714) (2) which placed *A3Bi* downstream of a *loxP*-flanked transcription stop cassette (**Supplementary Fig. 2A**). The functionality of this construct was confirmed by directional subcloning into the *Nhe*I/*Cla*I sites of pcDNA3.1 (ThermoFisher) followed by co-transfecting 293T cells with a control vector or Cre recombinase expression plasmid (TransitLT-1, Mirus) and, 48h later, performing *A3B*-specific RT-qPCR, anti-A3B immunoblots, and single-stranded DNA C-to-U activity assays (procedures described below; **Supplementary Fig. 2B, C**). Additional experiments showed that the hallmark nuclear localization of human A3B in human cells (3–5) is also evident upon expression in murine NIH 3T3 cells (**Supplementary Fig. 2D**). 10μg of functionally confirmed targeting vector was linearized with *Sac*II and electroporated into C57BL/6N embryonic stem (ES) cells (Ingenious Targeting Laboratory). G418 was used to select clones and PCR screening was used to identify homologous recombinants (upstream forward primer RSH8979 5'-ACT-GAC-CGC-ACG-GGG-ATT-CC and *neo* reverse primer RSH8985 5'-ACT-GAC-CGC-ACG-GGG-ATT-CC). Positive ES clones were confirmed by digesting genomic DNA with *Mfe*I and *Bsr*G1, separating on a 0.8% agarose gel, transferring to nylon membrane, and Southern blotting (restriction sites and probe locations shown in **Supplementary Fig. 2A** and blot images in **Supplementary Fig. 2E, F**). Correctly targeted ES clone 243 was microinjected into BALB/c blastocysts, and chimeric pups with high percentages of black coat colour were mated to wildtype C57BL/6J mice to obtain germline transmission.

Animal husbandry and genotyping

Mice were maintained at the University of Minnesota in AAALAC-accredited animal facilities (IACUC protocol 1302A30327). Heterozygous *Rosa26::LSL-A3Bi* (2x *loxP*) female mice were crossed with CMV-Cre male animals to remove the transcription stop cassette and obtain *Rosa26::L-A3Bi* (1x *loxP*) progeny (Jackson Lab Stock No: 006054 provided by Yasuhiko Kawakami, University of Minnesota). Cre-mediated recombination was verified by a PCR-based strategy (**Supplementary Fig. 2G**; methods detailed below). Because the CMV-Cre transgene is located on the X-chromosome, *Rosa26::L-A3Bi* (1x *loxP*) males were bred with wildtype females to generate a cohort with whole-body A3B expression. Human A3B expression was demonstrated in multiple tissues by RT-qPCR and single-strand DNA deaminase activity assays (**Supplementary Fig. 2H**). Protein-level expression and nuclear localization was also demonstrated by immunofluorescent microscopy of A3B in MEFs and anti-A3B IHC of a wide range of tissues including lung (procedures described below and representative data in **Supplementary Fig. 2I, J**. Expression of human A3B from the *Rosa26* promoter did not compromise fertility, as evidenced by normal Mendelian progeny ratios (not shown). Moreover, whole-body expression of A3B caused no overt somatic phenotypes in otherwise wildtype C57BL/6J animals. Mice were monitored weekly for tumour formation and euthanized by CO_2 asphyxiation upon natural health decline. The overall life expectancy of animals with whole-body expression of A3B was indistinguishable from animals without Cre-mediated induction of the minigene (**Supplementary Fig. 2K**). Moreover, rates of tumour occurrence were similar between the two groups (**Supplementary Fig. 2L**). Tumours were rare and average tumour numbers were similarly low in A3B-expressing versus non-expressing controls (**Supplementary Fig. 2M**). Genomic DNA was isolated from tail biopsies of 21-day-old mice using the Gentra Puregene protocol (Qiagen) and 50ng was used as a template for each diagnostic PCR reaction. The *Rosa26::LSL-A3Bi* (unrecombined 2x *loxP* allele) was detected by PCR of a 500 bp fragment using primers RSH8980 5'-AGC-ACT-TGC-TCT-CCC-AAA-GTC (*Rosa26* forward) and RSH8985 and 5'-TGC-GAG-GCC-AGA-GGC-CAC-TTG-TGT-AGC (LSL cassette reverse). The *Rosa26::L-A3Bi* (recombined 1x *loxP* allele) was detected by PCR of a 598bp fragment using the same *Rosa26* forward primer and RSH8984 5'-GCA-CAT-TTC-TGC-GTG-GTA-CTG-AGG (A3B reverse). The wildtype *Rosa26* locus was detected as a 300bp PCR product using the same *Rosa26* forward primer and RSH10347 5'-CAC-CTG-TTC-

AAT-TCC-CCT-GC-3' (*Rosa26* reverse). CMV-Cre was detected by standard PCR of a 100bp fragment using primers 5'-GCG-GTC-TGG-CAG-TAA-AAA-CTA-TC (RSH7141) and 5'-GTG-AAA-CAG-CAT-TGC-TGT-CAC-TT (RSH7142). A representative agarose gel image is shown in **Supplementary Fig. 2g**.

Cell culture

293T, NIH 3T3, and primary MEFs were cultured at 37°C containing 5% CO_2 and maintained in Dulbecco's Modified Eagle Media (DMEM) supplemented with 10% fetal bovine serum (FBS), 100U/mL penicillin, 100µg/mL streptomycin. ES cells were plated on irradiated primary MEF feeder cells plated on 0.1% gelatin coated plates. ES cells were cultured at 37°C containing 7.5% CO_2 and fed daily with IMDM supplemented with 15% FBS, 1x sodium pyruvate (Invitrogen), 1x L-glutamine (Invitrogen), 1x non-essential amino acids (Sigma-Aldrich), 100U/mL penicillin, 100µg/mL streptomycin, 10ng/ml LIF (Millipore), and 1x 2-mercaptoethanol (ThermoFisher Scientific).

RT-qPCR for quantification of *A3B* mRNA levels

Total RNA was isolated with the Qiagen RNAeasy protocol with QIAshredder and on column DNase treatment. cDNA was prepared and *A3B* mRNA expression was quantified by RT-qPCR as described(5). *A3B* expression was quantified using a LightCycler 480 (Roche) with forward primer 5'-GAC-CCT-TTG-GTC-CTT-CGA-C (RSH3220) and reverse primer 5'-GCA-CAG-CCC-CAG-GAG-AAG (RSH3221) and detected using Roche UPL #01. The housekeeping gene, human *TBP* or murine *Tbp*, was used for gene expression normalization between samples. Human *TBP* was amplified using primers 5'-CCC-ATG-ACT-CCC-ATG-ACC (RSH3231) and 5'-TTT-ACA-ACC-AAG-ATT-CAC-TGT-GG (RSH3232) and detected using Roche UPL #51. Murine *Tbp* was amplified using primers 5'- GGG-GAG-CTG-TGA-TGT-GAA-GT (RSH2913) and 5'- CCA-GGA-AAT-AAT-TCT-GGC-TCA (RSH2914) and detected using Roche UPL #97.

A3B immunoblots and single-stranded DNA C-to-U activity assays

Anti-A3B immunoblots were performed using established procedures (6–9). Whole cell extracts were prepared by boiling cell suspensions for 30 min in 2.5x reducing sample buffer (31mM Tris pH6.8, 10% glycerol, 1% SDS, 1.25% 2-mercaptoethanol, and 0.05% bromophenol blue). Soluble proteins from 30,000 cells were fractionated

by SDS-PAGE (12.5% polyacrylamide gel), transferred to PVDF membrane, and probed with the rabbit anti-human A3B mAb 5210-87-13 (1:1000; Harris lab custom reagent) (6). A murine anti-alpha tubulin mAb was used as a loading control (1:40,000; clone B-5-1-2, Sigma T5168). Licor secondary antibodies, anti-rabbit IRdye 800CW (Licor 827-08365) and anti-mouse IRdye 680LT (Licor 925-68020), were used at 1:20,000 and visualized using a Licor Odyssey imaging system.

Cell extracts were prepared for single-stranded DNA C-to-U activity assays as described (7–10). Cells were suspended in HED buffer (25mM HEPES, 5mM EDTA, 10% glycerol, 1mM DTT, 1 tablet proteasome inhibitor (Roche) per 50 ml buffer), freeze/thawed once, and vortexed to promote lysis. Cell debris was removed by centrifugation and cleared lysates were incubated 2h with 4pmol of a fluorescently labelled ssDNA substrate with a single target cytosine (5'-ATT-ATT-ATT-ATT-CGA-ATG-GAT-TTA-TTT-ATT-TAT-TTA-TTT-ATT-T-fluorescein; RSH5195). These conditions support A3B dependent ssDNA cytosine deamination and excision of the resulting uracil by uracil DNA glycosylase. Cleavage of uracil-excised substrates was promoted by adding NaOH to a final concentration of 0.1M and incubating samples at 98°C for 5 min. The resulting samples were fractionated on a 15% acrylamide gel, imaged (Typhoon, GE Healthcare Life Sciences), and quantified by densitometry (ImageQuant).

A3B immunohistochemistry (IHC)

IHC staining was performed following described procedures (6,11,12) FFPE tissues were sectioned at 4µm, mounted on positively charged, adhesive slides and allowed to air-dry for at least 24h. To deparaffinize and rehydrate the samples, slides were baked in a 65°C oven for 20 min, washed 3 times with CitrisolvTM (Decon Labs, #1601) for 5-min/each, soaked in graded alcohols (100% x 2, 95% and 80% for 3 min/each), and then rinsed in running water for at least 5 min. Epitope retrieval was performed using Reveal Decloaker (BioCare Medical, #RV1000M) in a steamer for 35 min, followed by a 20 min "cool-down" period. Then, slides were rinsed with running tap water for 5 min and transferred to TBST for 5 min. Endogenous peroxidase activity was quenched by placing the slides in 3% H2O2 in TBST for 10 min at RT, followed by a 5-min rinse under running water. To block non-specific binding of primary antibody, sections were covered with Background Sniper (BioCare Medical, #BS966MM) for 15 min at RT. After blocking, serial sections of each specimen were

incubated overnight at 4°C with a rabbit anti-human A3B mAb (5210-87-13) (6) diluted 1:350 in 10% Sniper in TBST. This mAb does not cross-react with murine A3.

Following overnight incubation with primary antibody, sections were rinsed in TBST for 5 min, and completely covered with anti-rabbit poly-HRP-IgG (Leica Biosystems, Novolink Polymer, #RE7260-K) for 30 min at RT. The reaction product was developed using the Novolink DAB substrate kit (Leica Biosystems, # RE7230-K) at RT for 3-5 min, rinsed in tap water for 5 min, counterstained in Mayer's hematoxylin solution (Electron Microscopy Sciences, #26252-01) for up to 5 min, dehydrated in graded alcohols and CitrisolvTM, and cover-slipped using Permount mounting media. The stained slides were scanned at 40x magnification and A3B nuclear immunoreactivity was visualized with the Aperio ScanScope XT (Leica Biosystems).

Gene editing

Double-stranded DNA breaks were induced at the *Kras* locus in the KPB6 cell line by nucleofection (Amaxa Nucleofector kit V, protocol T-013) of the pX458 vector, as previously described (13) using the following sgRNA: 5'-CTTGTGGTGGTTGGAGCT GA-3'. Homologous recombination repair was induced using the following single-stranded DNA template: 5'-ATTTAGTTGTATTTTATTATTTTTATTGTAAGGCCTG CTGAAAATGACTGAGTATAAGCTAGTCGTATTGGAGCTTGTGGCGTAGGCAA GAGCGCCTTGACGATACAGCTAATTCAGAATCACTTTGTGGATGAA-3'0.

Prime editing technology was used to induce a single-stranded DNA break and reverse transcriptase-mediated genome editing in KPAR1.3 cells, as previously described (14). Golden Gate Assembly was used to clone the sgRNA (5'TCAGCTCCA ACCACCACAAG-3'), 3' extension sequence: (GCCTACGCCACAAGCTCCAACC ACCA-3') and sgRNA scaffold (5'-CTAGAAATAGCAAGTTAAAATAAGGCTAG TCCGTTATCAACTTGAAAAAGTGGCACCGAGTCG-3') into a mammalian U6 expression vector (Addgene, #132777). To increase the efficiency of editing, a second sgRNA lacking the 3' extension sequence and targeting upstream of the editing point was generated by cloning the following sgRNA: 5'-TATACTCAGTCAT TTTCAGC-3' and identical sgRNA scaffold sequence into the U6 vector. Guide-expressing plasmids and a Cas9-H840A/GFP-expressing plasmid (Addgene, #132776) were transfected into KPAR1.3 cells by nucleofection (Amaxa Basic Nucleofector Kit for Primary Mammalian Epithelial Cells, protocol T-030). All DNA oligos were ordered annealed

and phosphorylated from Integrated DNA Technologies and overhang sequences were included to make them suitable for Golden Gate assembly.

For both KPAR1.3 and KPB6 cells, genomic DNA from single cell clones was extracted using QuickExtract DNA Extraction Solution (Lucigen) and amplified with PrimeSTAR Max DNA polymerase (Takara Bio) using primers Kras-F: 5'-GTCC ACAGGGTATAGCGTACT-3' and Kras-R 5'-CACCCAGTTTAAAGCCTTGGA-3'. PCR products were digested for 1h at 37°C with BfaI (NEB) for the KPB6 cell line or BccI (NEB) for KPAR1.3 cells. Digestion products were evaluated by gel electrophoresis with a 2% agarose gel.

Genotypes of single-cell clones were verified by Sanger sequencing using the following primer: 5'-CACCCAGTTTAAAGCCTTGGA-3'. Alternatively, Illumina MiSeq next-generation sequencing was used. For this purpose, genomic DNA was amplified with CloneAmp HiFi PCR Premix (Takara Bio) using primers Kras1-F: 5'-TCGTCGGCAGCGTCAGATGTGTATAAGAGACAGGTCCACAGGGTATAGCGT ACT-3' and Kras1-R: 5'-GTCTCGTGGGCTCGGAGATGTGTATAAGAGACAGTTAC AAGCGCACGCAGACT-3'. PCR products were purified using AMPure XP beads according to the manufacturer's instructions and sequenced on the Illumina MiSeq. All sequences were visualized and analysed using SnapGene Software.

Emv2$^{-/-}$ KPAR cell lines were generated by transient transfection of a Cas9-sgRNA plasmid (pX458, Addgene) generated by standard molecular cloning techniques using the following sgRNA: 5'- AAAGGCTTTATTGGATACAC-3' targeting the LTR sequence of the *Emv2* locus. Cells were transfected and after 24h GFP-positive cells were single-cell cloned by FACS sorting. Clones were assessed for loss of eMLV envelope protein surface expression by flow cytometry to identify KO cell lines.

Supplementary References

1. Hultquist JF, Lengyel JA, Refsland EW, LaRue RS, Lackey L, Brown WL, et al. Human and Rhesus APOBEC3D, APOBEC3F, APOBEC3G, and APOBEC3H Demonstrate a Conserved Capacity To Restrict Vif-Deficient HIV-1. J Virol. 2011;85:11220–34.
2. Soriano P. Generalized lacZ expression with the ROSA26 Cre reporter strain. Nat Genet [Internet]. 1999;21:70–1. Available from: https://www.nature.com/articles/ng0199_70
3. Stenglein MD, Harris RS. APOBEC3B and APOBEC3F inhibit L1 retrotransposition by a DNA deamination-independent mechanism. J Biol Chem. 2006;281:16837–41.
4. Lackey L, Demorest ZL, Land AM, Hultquist JF, Brown WL, Harris RS. APOBEC3B and AID have similar nuclear import mechanisms. J Mol Biol [Internet]. Elsevier Ltd; 2012;419:301–14. Available from: http://dx.doi.org/10.1016/j.jmb.2012.03.011
5. Salamango DJ, McCann JL, Demir Ö, Brown WL, Amaro RE, Harris RS. APOBEC3B Nuclear Localization Requires Two Distinct N- Terminal Domain Surfaces. Physiol Behav. 2018;176:139–48.
6. Brown WL, Law EK, Carpenter MA, Argyris PP, Levin-Klein R, Ranum AN, et al. A rabbit monoclonal antibody against the antiviral and cancer genomic DNA mutating enzyme APOBEC3B. Antibodies (Basel). 2019;8.
7. Olson ME, Li M, Harris RS, Harki DA. Small-Molecule APOBEC3G DNA Cytosine Deaminase Inhibitors Based on a 4-Amino-1,2,4-triazole-3-thiol Scaffold. ChemMedChem. 2013;8:112–7.
8. Leonard B, McCann JL, Starrett GJ, Kosyakovsky L, Luengas EM, Molan AM, et al. The PKC/NF-κB signaling pathway induces APOBEC3B expression in multiple human cancers. Cancer Res. 2015;75:4538–47.
9. Law EK, Levin-Klein R, Jarvis MC, Kim H, Argyris PP, Carpenter MA, et al. APOBEC3A Catalyzes Mutation and Drives Carcinogenesis In Vivo. J Exp Med. 2020;217.
10. Burns MB, Lackey L, Carpenter MA, Land AM, Leonard B, Refsland EW, et al. APOBEC3B is an enzymatic source of mutation in breast cancer. Nature. 2014;494:366–70.
11. Argyris PP, Wilkinson PE, Jarvis MC, Magliocca KR, Patel MR, Vogel RI, et al. Endogenous APOBEC3B overexpression characterizes HPV-positive and HPV-negative oral epithelial dysplasias and head and neck cancers. Mod Pathol [Internet]. Springer US; 2020; Available from: http://dx.doi.org/10.1038/s41379-020-0617-x
12. Serebrenik AA, Argyris PP, Jarvis MC, Brown WL, Bazzaro M, Vogel RI, et al. The DNA Cytosine Deaminase APOBEC3B is a Molecular Determinant of Platinum Responsiveness in Clear Cell Ovarian Cancer. Clin Cancer Res. 2020;26:3397–407.
13. Ran FA, Hsu PD, Wright J, Agarwala V, Scott DA, Zhang F. Genome engineering using the CRISPR-Cas9 system. Nat Protoc. 2013;8:2281–308.
14. Anzalone A V., Randolph PB, Davis JR, Sousa AA, Koblan LW, Levy JM, et al. Search-and-replace genome editing without double-strand breaks or donor DNA. Nature [Internet]. Springer US; 2019;576:149–57. Available from: http://dx.doi.org/10.1038/s41586-019-1711-4

Supplementary figure 1

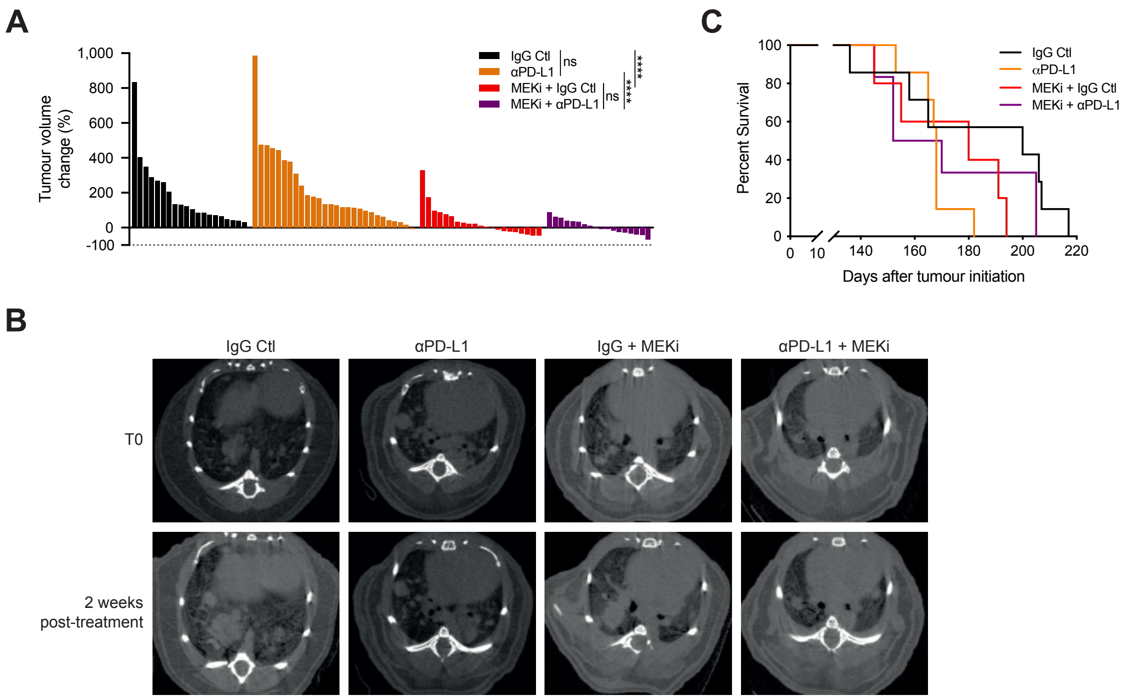

Supplementary figure 1. MEK inhibition does not improve response of KP tumours to anti-PD-L1
(A) Waterfall plot of tumour volume change in KP-tumour-bearing mice treated intraperitoneally with anti-PD-L1 (10mg/kg) or corresponding isotype control (IgG Ctl) twice weekly for 2 weeks and/or trametinib (3mg/kg daily oral gavage). Treatment began 16 weeks after tumour initiation. Each bar represents volume change in a single tumour, n=5-7 mice per group. One-way ANOVA; ns $P>0.05$, **** $P\leq 0.0001$.
(B) Representative micro-CT scans of mice treated as in (A).
(C) Survival of KP-tumour-bearing mouse treated as in (A).

Supplementary figure 2

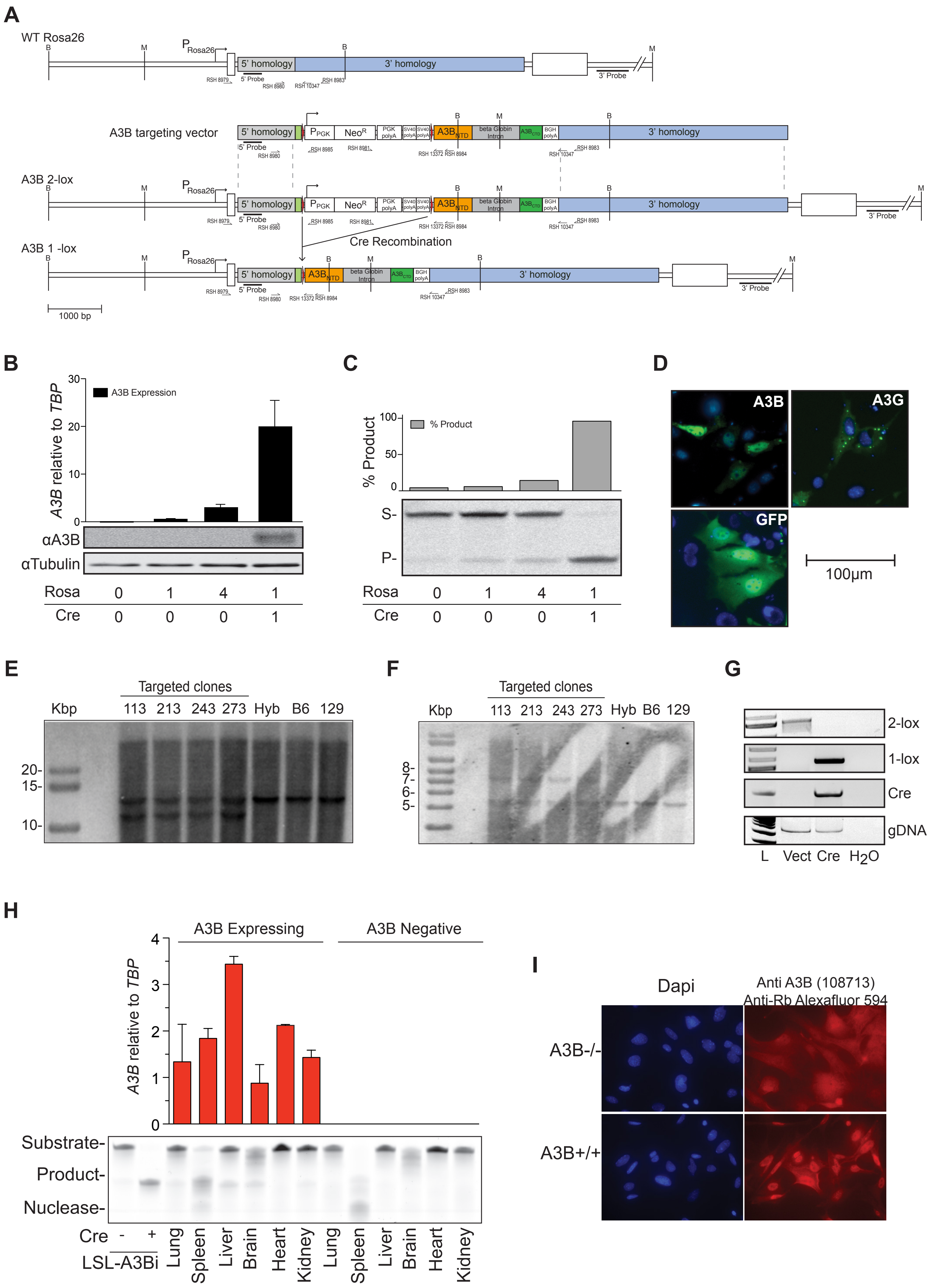

Supplementary figure 2 continue

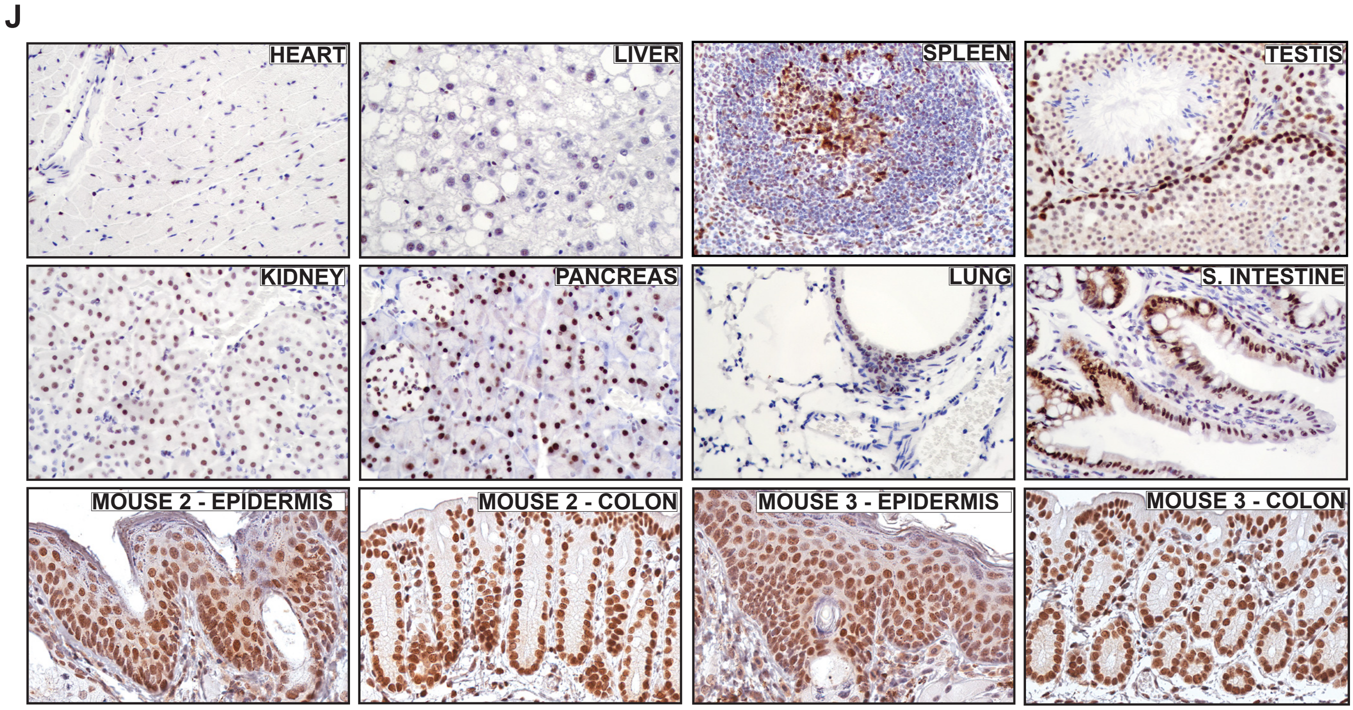

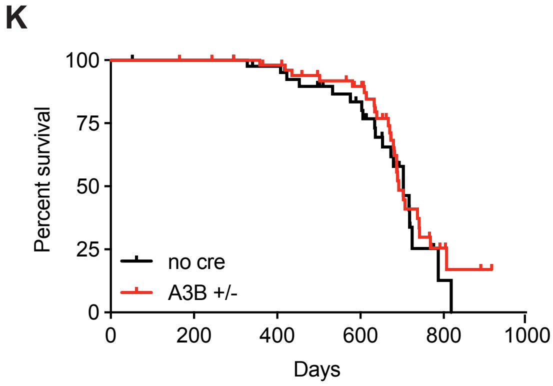

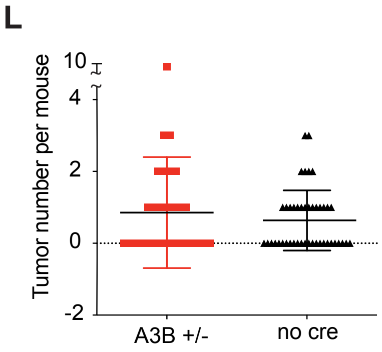

Supplementary figure 2 continued

Supplementary figure 2. Creation and validation of a Cre-inducible human A3B minigene for whole-body or tissue-specific expression in mice

(A) Schematic of the wild-type murine Rosa26 locus, the LSL-A3Bi gene targeting construct with 5' and 3' homology arms, the targeted locus after homologous recombination (Rosa26::LSL-A3Bi), and the targeted locus after Cre-mediated induction of A3Bi minigene expression (Rosa26::L-A3Bi).

(B) Detection of A3B mRNA (top) and protein (bottom) levels in 293T cells following co-transfection with pcDNA3.1-LSL-A3Bi in the absence or presence of Cre recombinase. The numbers indicate microgram amounts of transfected plasmid.

(C) Quantification (top) of single-stranded DNA C-to-U activity (bottom) in 293T extracts after co-transfection with pcDNA3.1-LSL-A3Bi in the absence or presence of Cre recombinase (S, substrate; P, product). The numbers indicate microgram amounts of transfected plasmid.

(D) Fluorescent microscopy images of NIH 3T3 cells transfected with the indicated eGFP-tagged constructs. A3B shows characteristic nuclear localization, A3G is cytoplasmic, and the eGFP control is cell wide. Nuclei are shown in blue (0.01% Hoechst 33342). The scale bar indicates 100μm.

(E-F) Southern blot analysis of genomic DNA from the indicated PCR-positive ES clones and controls [B6/129 hybrid untargeted ES cells (Hyb), C57BL/6 untargeted ES cells (B6), and SV129 untargeted ES cells (129)]. Genomic DNA was digested with MfeI or BsrGI, fractionated by agarose gel electrophoresis, transferred to membrane, and hybridized respectively with the 3' or the 5' probe depicted in panel a. The MfeI blot yields a 10.96 kbp band for correct targeting and a 12.61 kbp band for the untargeted Rosa26 locus. The BsrGI blot yields a 7.74 kbp band for correct targeting and a 5.61 kbp band for untargeted Rosa26 locus.

(G) Representative results from PCR-based genotyping assays. Non-Cre expressing conditions yield a 500 bp band characteristic of the non-recombined Rosa26::LSL-A3Bi minigene (2x loxP). Cre expression removes the LSL cassette and results in a single loxP site within a characteristic 598 bp PCR product (1x loxP). Specific bands for Cre (100 bp) and murine IL-2 (324 bp) are also shown.

(H) A3B mRNA (top) and DNA C-to-U activity (bottom) levels in the indicated tissues. DNA C-to-U activity in splenic tissue is frequently occluded by nucleolytic degradation of the substrate single-stranded DNA. Activity in heart was not detected due to poor tissue disruption and protein extraction.

(I) Immunofluorescent microscopy of MEFs from control embryos and Rosa26::L-A3Bi expressing embryos.

(J) Immunohistochemical detection of human A3B in the indicated tissues from Rosa26::L-A3Bi mice. The top 8 images are from a representative Rosa26::L-A3Bi male, and the bottom 4 are from two additional animals to demonstrate reproducibility. The characteristic nuclear localization of A3B is clear in all tissues and most cell types. A3B IHC signals are higher in some tissues in comparison to others, likely reflecting natural differences in Rosa26 promoter activity.

(K) Kaplan-Meier plots comparing rates of overall survival of a large cohort of Rosa26::L-A3Bi animals (n=53) and a similarly large control cohort with the non-Cre-induced minigene (n=45; log-rank test, p=0.61). Similar numbers of males and females were monitored and no differences were noted between the two sexes.

(L) A dot plot showing the numbers of tumours observed in necropsies of the groups described in panel K (Wilcoxon Rank Sum test, p=0.13)

Supplementary figure 3

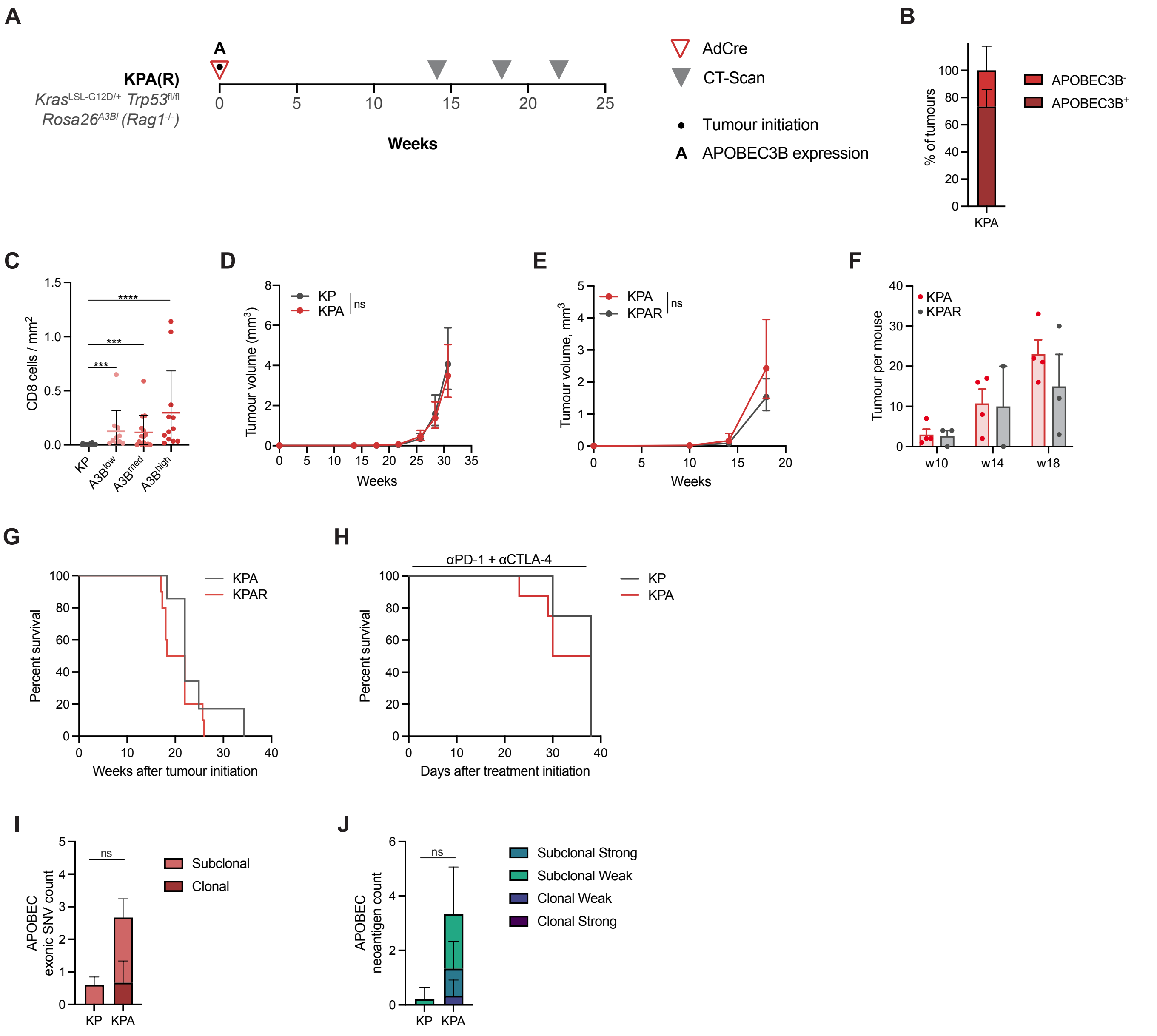

Supplementary figure 3. A3Bi expression in KP tumours

(A) Schematic of KPA tumour induction. Tumours were initiated and A3Bi expressed following AdCre intratracheal delivery in *Kras*$^{G-12D/+}$;*Trp53*$^{fl/fl}$;*Rosa26*^{A3Bi} (KPA) or *Kras*$^{G12D/+}$;*Trp53*$^{fl/fl}$;*Rosa26*^{A3Bi};*Rag1*$^{-/-}$ (KPAR) mice. The mice were regularly CT-scanned.

(B) Percentage of APOBEC3B positive and negative tumours in 30 KPA tumour from 5 mice, estimated by immunohistochemistry. Mean, upper and lower limit.

(C) Quantification of immunohistochemistry staining for CD8 in KP and KPA tumours. KPA tumours broken-down according to APOBEC3B expression. Mean per group, ±SD, n=5 mice per group. Kruskal-Wallis test, FDR 0.05; *** P≤0.001, **** P≤0.0001

(D) Tumour volume progression in KP (n=4 mice) and KPA (n=3 mice) models estimated by micro-CT scans. Geometric mean ± 95% CI. Two-way ANOVA, FDR 0.05; ns. P>0.05.

(E) Tumour volume progression in KPA (n=5 mice) and KPAR (n=3 mice) models estimated by micro-CT scans. Mean ±SEM. Two-way ANOVA, FDR 0.05; ns P>0.05.

(F) Number of tumours per mouse in KPA (n=5 mice) and KPAR (n=3 mice) models estimated from micro-CT scans.

(G) Survival of KPA- (n=6) and KPAR- (n=10) tumour-bearing mice.

(H) Survival of KP- (n=4) and KPA- (n=8) tumour-bearing mice treated with 200µg of anti-PD-1 and 200µg of anti-CTLA-4.

(I-J) Mean APOBEC-specific (T(C>T/G)) exonic SNV count ±SD (I) and neoantigen count ±SD (J) in KP (n=5 tumours) and KPA (n=3 tumours) broken down into clonal and subclonal. Unpaired, two-tailed Student's t-test performed on mean of all SNVs or neo-antigen count; ns P>0.05.

Supplementary figure 4

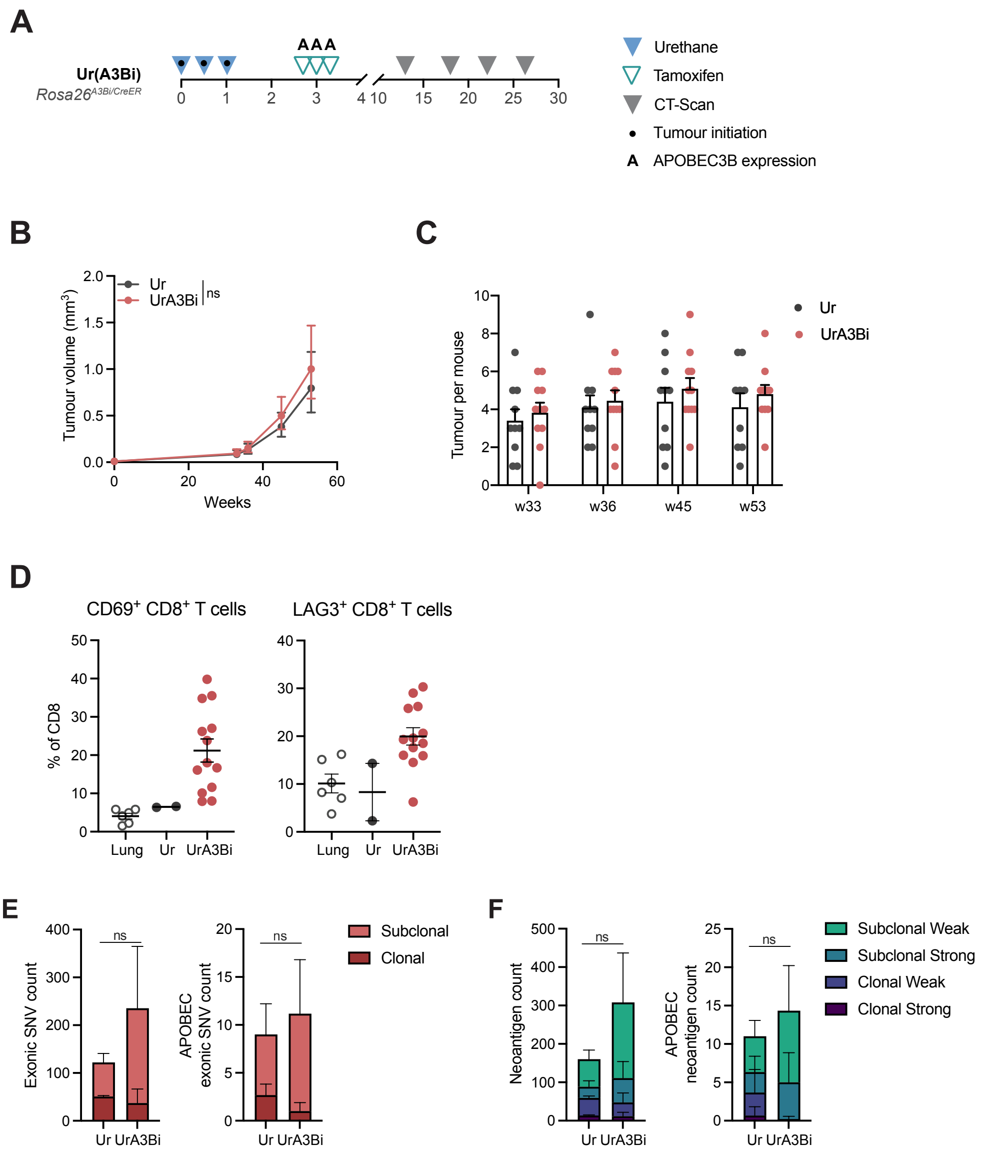

Supplementary figure 4 | A3Bi expression in urethane-induced tumours
(A) Schematic of UrA3Bi tumour induction. Tumours were initiated by 3 intra-peritoneal injections of urethane (1mg/g) and A3Bi expressed following 3 doses of tamoxifen (150mg/g) (UrA3Bi).The mice were regularly CT-scanned.
(B) Tumour volume progression in Ur (n=9 mice) and UrA3Bi (n=10 mice) models estimated by micro-CT scans. Data are mean tumour volumes ±SEM. Two-way ANOVA, FDR 0.05; ns P>0.05.
(C) Mean number of tumours per mouse in Ur (n=9 mice) and UrA3Bi (n=11 mice) models estimated from micro-CT scans.
(D) Frequency of CD69$^+$ (left) and LAG3$^+$ (right) CD8$^+$ T cells in Ur and UrA3Bi tumours compared to normal lung, determined by flow cytometry. Data are mean ± SEM, n=6 mice (normal lung), n=2 mice (Ur) or n=13 mice (UrA3Bi). Symbols represent pooled tumours from individual mice.
(E-F) Mean exonic SNVs count ±SD (E) and neoantigen count ±SD (F) in Ur (n=3 tumours) and UrA3Bi (n=6 tumours) broken down into clonal and subclonal. All (left panel) and APOBEC-specific (T(C>T/G)) (right panel). Unpaired, two-tailed Student's t-test performed on mean of all SNVs or neoantigen count; ns P>0.05. Peptides with a rank threshold of <2 or <0.5 were designated as weak or strong MHC-I binders, respectively.

Supplementary figure 5

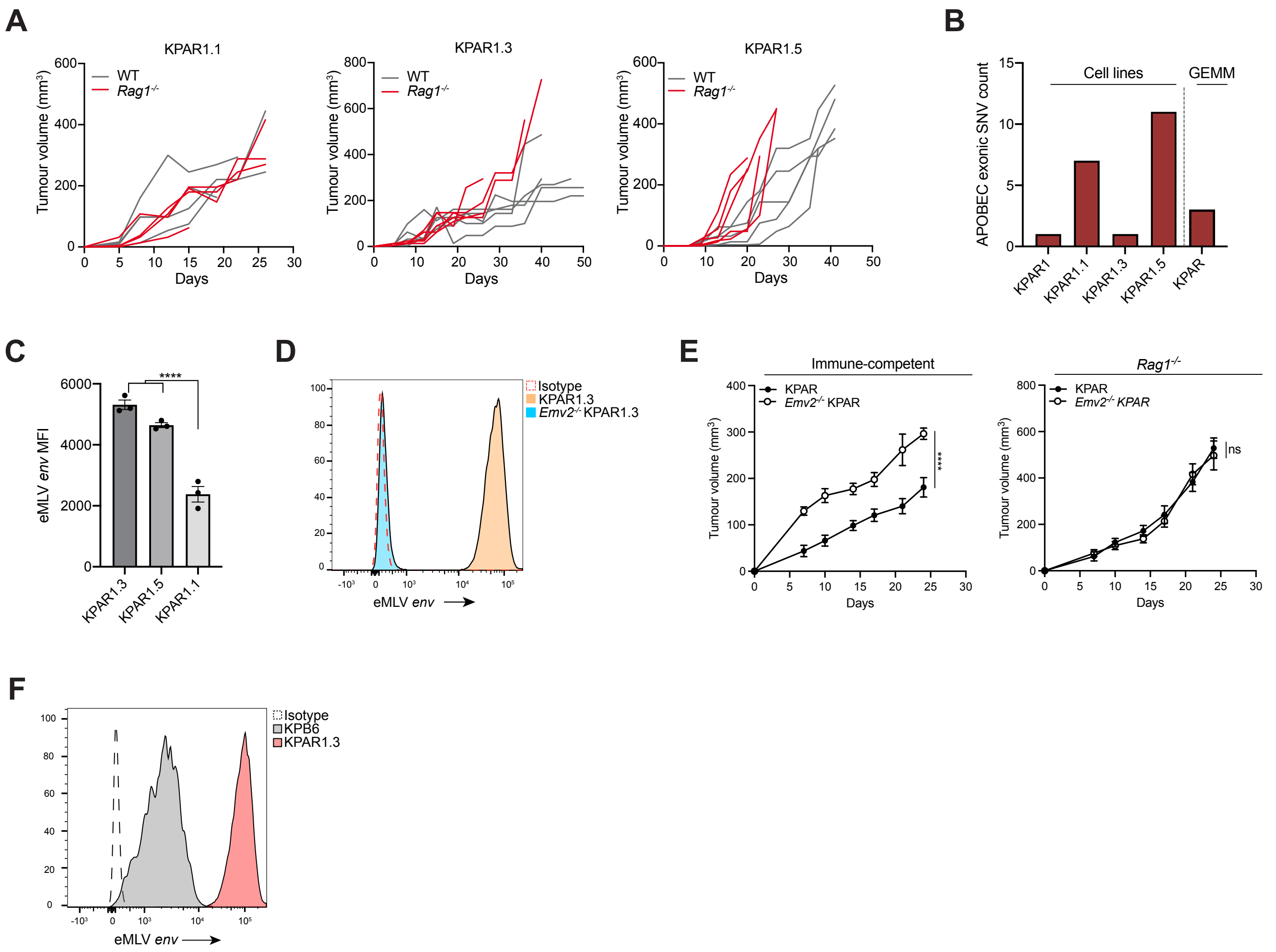

Supplementary figure 5. eMLV expression contributes to the immunogenicity of KPAR1.3 cells

(A) Growth of individual KPAR subclone tumours in immune-competent and *Rag1*$^{-/-}$ mice. n=4-5 mice per group.

(B) Frequency of APOBEC-specific (T(C>T/G)) exonic mutations in an autochthonous KPAR tumour, the KPAR parental cell line and the KPAR1.1, KPAR1.3 and KPAR1.5 single-cell clones, estimated by whole-exome sequencing.

(C) Surface expression of eMLV envelope glycoprotein on KPAR1.1, KPAR1.3 and KPAR1.5 cells. Data are mean ± SEM. One-way ANOVA; **** P≤0.0001.

(D) Representative histogram plot of eMLV envelope expression on parental KPAR1.3 cells and *Emv2*$^{-/-}$ KPAR1.3 cells.

(E) Growth of parental KPAR1.3 and *Emv2*$^{-/-}$ KPAR1.3 subcutaneous tumours in immune-competent and *Rag1*$^{-/-}$ mice. Data are mean tumour volumes ± SEM, n=7-8 mice per group. Two-way ANOVA; ns P>0.05, **** P≤0.0001.

(F) Representative histogram plot of eMLV envelope expression on KPAR1.3 cells and KPB6 cells.

Supplementary figure 6

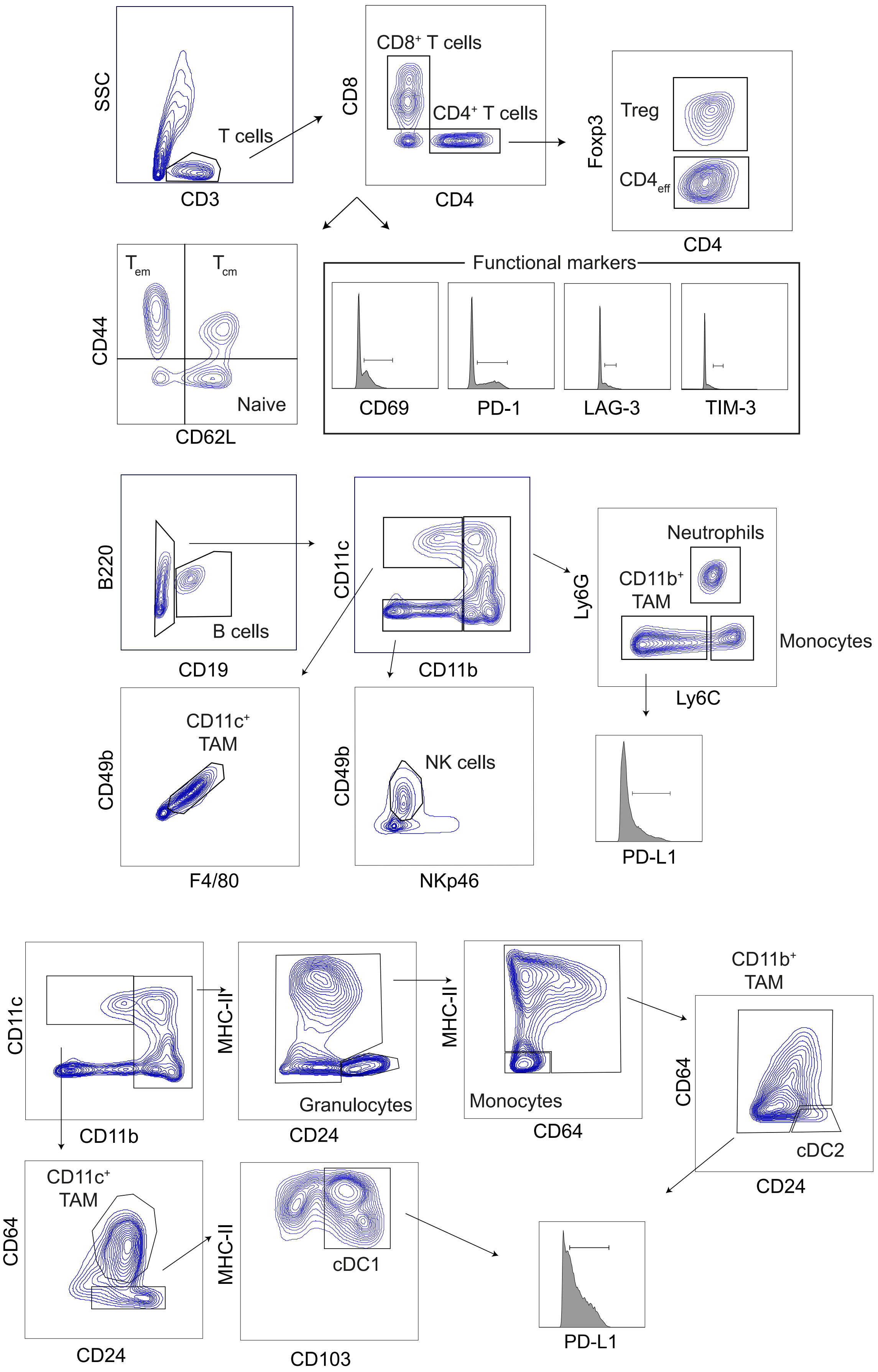

Supplementary figure 6. Flow cytometry characterisation of the tumour microenvironment
Representative flow cytometry plots after gating on $CD45^+$ live cells showing flow cytometry gating strategy.

Supplementary figure 7

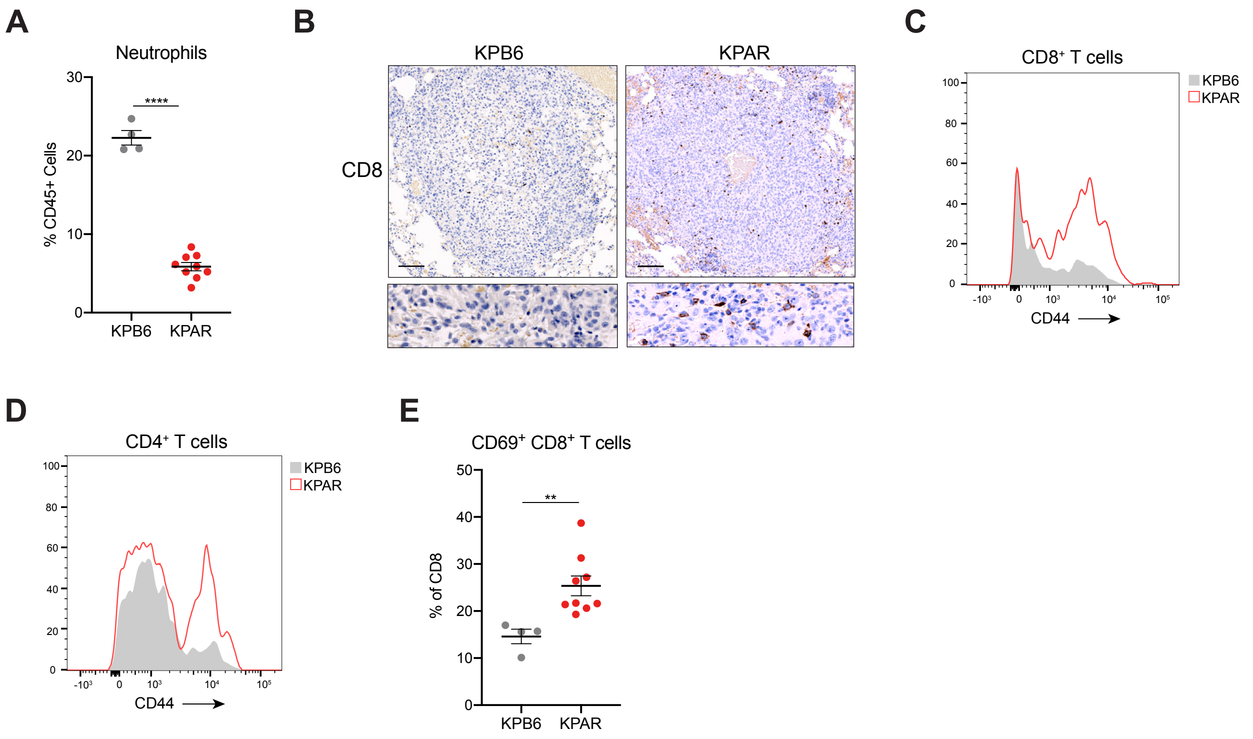

Supplementary figure 7. Flow cytometry analysis of KPAR and KPB6 orthotopic lung tumours
(A) Frequency of neutrophils in KPAR and KPB6 orthotopic tumours. Data are mean ± SEM. Unpaired, two-tailed Student's t-test; **** P≤0.0001.
(B) Representative immunohistochemistry staining for CD8 in KPAR and KPB6 orthotopic tumours. Scale bar represents 100μm.
(C-D) Representative histogram plots of CD44 surface expression on $CD8^+$ (C) and $CD4^+$ (D) T cells.
(E) Percentage of $CD69^+$ $CD8^+$ T cells. Data are mean ± SEM. Unpaired, two-tailed Student's t-test; ** P≤0.01.
Tumours were analysed 21 days after transplantation.

Supplementary figure 8

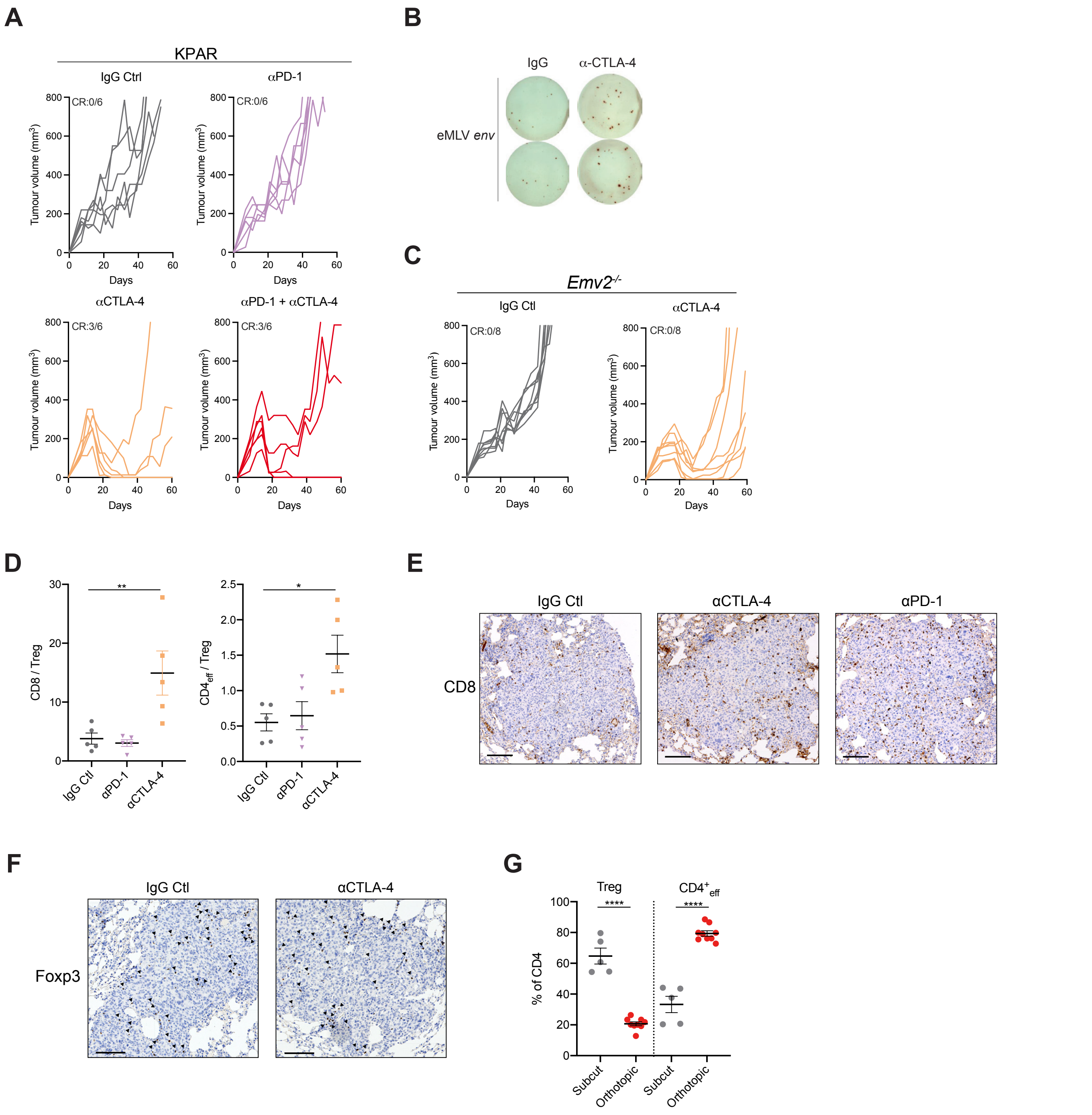

Supplementary figure 8. Response to immune checkpoint blockade is affected by the site of tumour growth

(A) Individual subcutaneous KPAR tumour volumes in mice treated intraperitoneally with 200μg anti-PD-1 and/or 200μg anti-CTLA-4 or corresponding isotype control (IgG Ctl) on day 10, 14, 17 and 21. CR, complete regression. n=6 mice per group.

(B) IFNγ ELISPOT analysis of TILs isolated from subcutaneous KPAR tumours treated with anti-CTLA-4 or isotype control as in (A). Treatment was on day 10, 14 and 17 and mice were culled on day 18. TILs were pooled from 6 mice per group and pulsed with eMLV *env* peptide.

(C) Individual subcutaneous *Emv2*$^{-/-}$ KPAR tumour volumes in mice (from Supplementary Fig. 5E) treated with anti-CTLA-4 as in (A). CR, complete regression. n=8 mice per group.

(D) Ratio of CD8$^+$ and CD4$^+$ T cells to Foxp3$^+$ Tregs in subcutaneous tumours treated as in (A). Treatment was on day 10, 14 and 17 and mice were culled on day 18. Data are mean ± SEM, n=5 mice per group. One-way ANOVA; * P≤0.05, ** P≤0.01.

(E) Representative immunohistochemistry staining for CD8 in orthotopic KPAR lung tumours treated intraperitoneally with 200μg anti-PD-1, 200μg anti-CTLA-4 or corresponding isotype controls twice weekly for two weeks. Treatment was initiated once tumours were detectable by micro-CT. Scale bar represents 100μm.

(F) Representative immunohistochemistry staining for Foxp3 in orthotopic KPAR lung tumours treated as in (E). Scale bar represents 100μm.

(G) Frequency of Foxp3$^+$ Tregs and CD4$_{eff}$ T cells in subcutaneous and orthotopic tumours. Data are mean ± SEM, n=5 mice (subcutaneous) or n=9 mice (orthotopic). Unpaired, two-tailed Student's t-test; **** P≤0.0001.

Supplementary figure 9

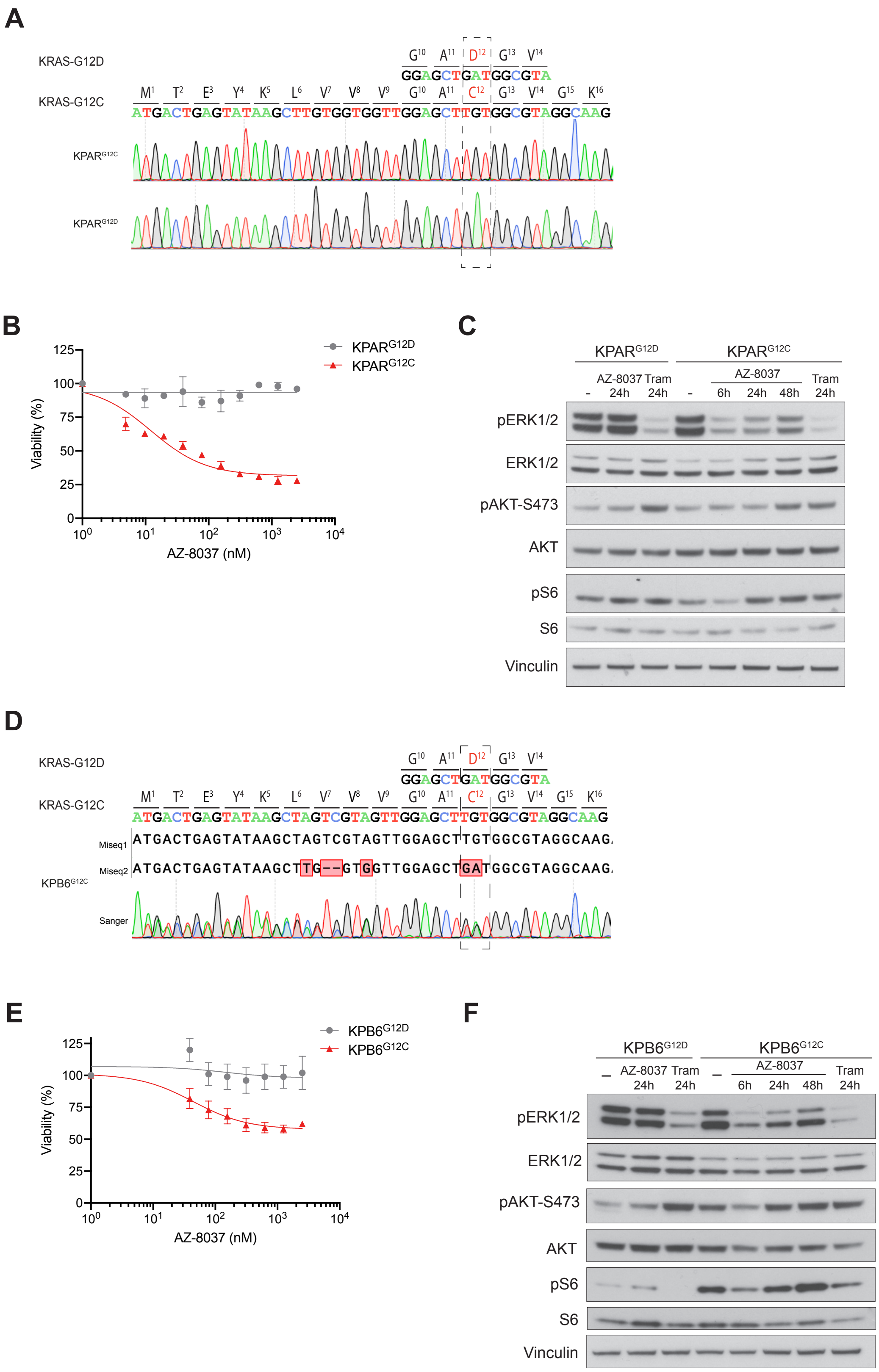

Supplementary figure 9. Generation of KPAR^{G12C} and KPB6^{G12C} cell lines
(A) Sanger sequencing chromatograms showing base-editing of both KRAS^{G12D} alleles of the KPAR cell line into KRAS^{G12C} alleles.
(B) Viability of parental KPAR and KPAR^{G12C} cells treated with serial dilutions of AZ-8037 for 72h. Data are mean ±SEM of two independent experiments.
(C) Western blot of parental KPAR and KPAR^{G12C} cells treated with 250nM AZ-8037 for 6h, 24h and 48h. Cells were treated with 10 nM trametinib (Tram) for 24h as a control.
(D) Sanger sequencing chromatogram and Miseq sequences showing knock-in of the KRAS^{G12D} allele into a KRAS^{G12C} allele (Miseq1) and knock-out of the wildtype allele (Miseq2) in KPB6^{G12C} cells.
(E) Viability of parental KPB6 and KPB6^{G12C} cells treated as in (B). Data are mean ±SEM of four independent experiments.
(F) Western blot of parental KPB6 and KPB6^{G12C} cells treated as in (C).

Supplementary figure 10

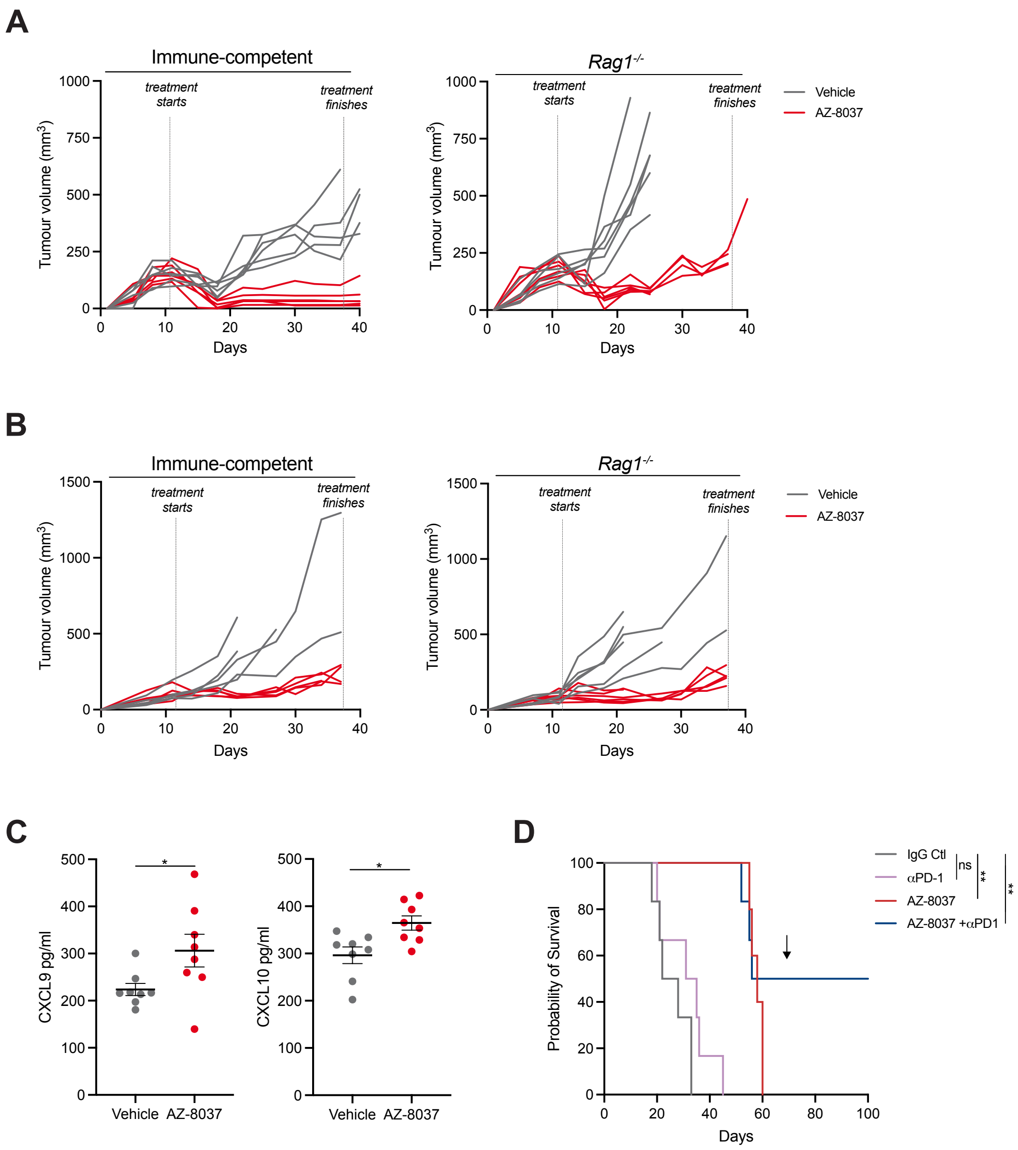

Supplementary figure 10. KRAS^{G12C} inhibition synergises with adaptive immunity and ICB
(A-B) Individual volumes of KPAR^{G12C} (A) and KPB6^{G12C} (B) subcutaneous tumours growing in immune-competent (left) and *Rag1*$^{-/-}$ (right) mice treated with vehicle or AZ-8037 for stated duration (100mg/kg daily oral gavage). n=6 mice per group.
(C) Concentration of CXCL9 and CXCL10 as determined by ELISA from KPAR^{G12C} tumours treated for 7 days with vehicle or AZ-8037.
(D) Kaplan-Meier survival of mice bearing KPAR^{G12C} subcutaneous tumours treated with AZ-8037 and/or 200µg anti-PD-1 or corresponding isotype control. AZ-8037 was administered daily for 4 weeks from day 14 and anti-PD-1 was administered on day 15, 18, 22 and 25. The black arrow indicates the time at which mice that previously rejected the primary tumour were re-challenged on the opposite flank. Log-rank (Mantel-Cox) test; ns P>0.05, ** P≤0.01.
